# Association of acute inflammatory cytokines, fracture malreduction, and functional outcome 12 months after intra-articular ankle fracture—a prospective cohort study of 46 patients with ankle fractures

**DOI:** 10.1186/s13018-021-02473-8

**Published:** 2021-05-25

**Authors:** That Minh Pham, Emil Bjoertomt Kristiansen, Lars Henrik Frich, Kate Lykke Lambertsen, Søren Overgaard, Hagen Schmal

**Affiliations:** 1 Department of Orthopedics and Traumatology, Odense University Hospital, J.B. Winsløws Vej 4, 5000 Odense, Denmark; 2 Department of Clinical Research, University of Southern Denmark, Odense, Denmark; 3 Department of Neurobiology Research, Institute of Molecular Medicine, University of Southern Denmark, Odense, Denmark; 4 Department of Neurology, Odense University Hospital, Odense, Denmark; 5 BRIDGE–Brain Research–Inter-Disciplinary Guided Excellence, Department of Clinical Research, University of Southern Denmark, Odense, Denmark; 6 Department of Clinical Medicine, Copenhagen University Hospital, Bispebjerg, Copenhagen, Denmark; 7 Clinic of Orthopedic Surgery, Medical Center—University of Freiburg, Faculty of Medicine, University of Freiburg, Freiburg, Germany; 8 OPEN, Odense Patient data Explorative Network, Odense University Hospital/Institute of Clinical Research, University of Southern Denmark, Odense, Denmark

**Keywords:** Inflammation, Osteoarthritis, Clinical outcomes, Quality of life, AOFAS, FFI, X-ray, Weight-bearing CT

## Abstract

**Background:**

Several malreduction criteria have been proposed for ankle surgery, but the criteria of most importance for functional outcome remain undetermined. Furthermore, the acute inflammatory response in the ankle joint after fracture is hypothesized to result in osteoarthritis development, but no study has investigated the correlation between the levels of these inflammatory cytokines and post-surgical functional outcomes. We aimed to identify malreduction criteria and inflammatory cytokines associated with functional outcome after ankle surgery.

**Methods:**

During surgery, synovial fluid from the fractured and healthy contralateral ankles of 46 patients was collected for chemiluminescence analysis of 22 inflammatory cytokines and metabolic proteins. The quality of fracture reduction was based on 9 criteria on plain X-rays and 5 criteria on weight-bearing computed tomography (WBCT) scans. After 3 and 12 months, we recorded scores on American Orthopedic Foot and Ankle Society (AOFAS) scale, the Danish version of Foot Function Index (FFI-DK), EQ-5D-5L index score, the Kellgren-Lawrence score, and joint space narrowing.

**Results:**

Tibiofibular (TF) overlap (*p* = 0.02) and dime sign (*p* = 0.008) correlated with FFI-DK. Tibiotalar tilt correlated positively with joint space narrowing at 3 months (*p* = 0.01) and 12 months (*p* = 0.03). TF widening correlated with FFI-DK (*p* = 0.04), AOFAS (*p* = 0.02), and EQ-5D-5L (*p* = 0.02). No consistent correlations between synovial cytokine levels and functional outcomes were found at 12 months.

**Conclusions:**

Malreduction of TF overlap, TF widening, and tibiotalar tilt were the most important criteria for functional outcome after ankle surgery. Increased inflammatory cytokine levels after fracture did not affect functional outcome at 12 months.

**Trial registration:**

This cohort study is registered the 10th of December 2018 at ClinicalTrials.gov (NCT03769909), was approved by the local committee on health ethics (The Regional Committees on Health Research Ethics for Southern Denmark: J.No. S-20170139), and was reported to the National Danish Data Protection Agency (17/28505).

**Supplementary Information:**

The online version contains supplementary material available at 10.1186/s13018-021-02473-8.

## Background

Ankle fracture is a serious joint injury as it can lead to severe complications, reduced function and quality of life, and permanent disability [1]. Up to 36% of intra-articular fractures in the lower extremity develop post-traumatic osteoarthritis (PTOA) [2, 3]. Besides high body mass index (BMI), age over 30 years, and fracture complexity, fracture malreduction during surgery has been reported to influence functional outcome and development of PTOA [4].

The current gold standard for treating unstable ankle fracture is surgery [1, 5]. Several radiographic parameters on plain x-ray and computed tomography (CT) have been proposed as criteria for a good fracture reduction [6]. No consensus has been reached, however, and the most important criteria for functional outcome after ankle surgery have not yet been determined [7]. In complex comminuted ankle fractures, it is not always possible for the surgeon to reduce the fracture perfectly during surgery, so it is crucial to identify the most important radiographic criteria for fracture reduction as well as their association with PTOA and functional outcome.

Additionally, recent studies implicate inflammatory cytokines in the development of PTOA [8, 9], and several studies report elevation of inflammatory cytokines in the joint space after acute intra-articular ankle fracture that can persist up to 6 months after surgery [10, 11]. To the best of our knowledge, however, no study has evaluated the correlation between cytokine levels post-injury and the functional outcome after surgery.

The aims of the current study were (1) to investigate whether malreduction criteria on X-ray and weight-bearing CT were associated with functional outcome 3 and 12 months after surgery, and (2) to identify the presence of inflammatory cytokines in the fractured ankle and to determine whether they were elevated post-injury and whether their levels were associated with functional outcome after ankle surgery.

## Methods

### Descriptive characteristics of the cohort

**Fig. 1:**
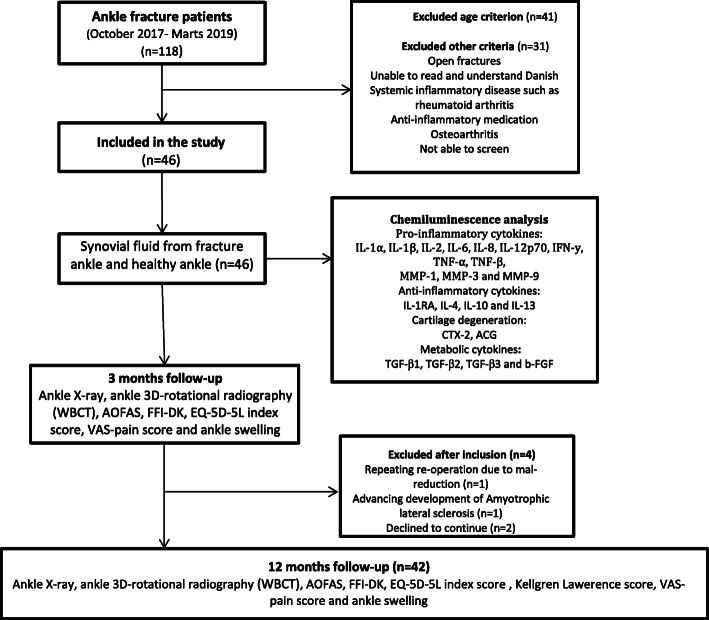
Flow diagram showing patient enrollment and study procedures

This cohort study was registered at ClinicalTrials.gov (NCT03769909) and approved by the National Committee on Health Research Ethics (J.No. S-20170139). The study is reported according to the STROBE guidelines (Additional file 1) [12]. Patients with acute intra-articular ankle fracture admitted to Odense University Hospital and Svendborg Hospital from October 2017 to March 2019 were enrolled in the study. Inclusion criteria were an acute intra-articular fracture involving the ankle joint, a need for internal or external fixation within 14 days, patient age between 18 and 65 years, ability to read and understand Danish, and written informed consent. Exclusion criteria were open fracture, associated arterial and nerve injury, multiple injury patients with an Injury Severity Score >15, primary or secondary infection, systemic inflammatory disease such as rheumatoid arthritis, anti-inflammatory medication, and injuries associated with a Charcot foot. Patients were also excluded if they had any sign of radiographic OA in the fractured or healthy contralateral ankle joint (Fig. 1).

The following epidemiological parameters were collected: age, sex, body mass index (BMI), American Society of Anesthesiologists Classification (ASA), and fracture classification of injury according to Arbeitsgemeinschaft für Osteosynthesefragen (AO) standards (44A, 44B, and 44C, time of samples collection, and surgical managements).

### Perioperative aspiration of synovial fluids for chemiluminescence analysis

Briefly, synovial fluid (SF) was collected prior to surgery from the healthy contralateral ankle joint and then the fractured joint by puncture using the antero-medial portal. As it can be difficult to obtain sufficient SF from the ankle joint [13], 5 ml saline was injected prior to aspiration. Within 2 h of sampling, SF samples were centrifuged at 2000 revolutions per minute (RPM) for 15 min, aliquoted, and stored at – 80 °C until chemiluminescence analysis.

### Chemiluminescence analysis of the synovial fluids

SF from the fractured and healthy contralateral ankle joints were analyzed for 12 pro-inflammatory and 4 anti-inflammatory cytokines, 4 metabolic proteins, and 2 cartilage degradation proteins.

SF levels of interleukin (IL)-1α, IL-1 receptor antagonist (IL-1RA), IL-1β, IL-2, IL-4, IL-6, IL-8, IL-10, IL-12p70, IL-13, interferon gamma (IFN-y), tumor necrosis factor (TNF)-α, and TNF-β were measured by an electrochemiluminescence immunoassay using a human customized U-Plex (Mesoscale, Rockville, MD). Matrix metalloproteinase (MMP)-1, MMP-3, and MMP-9 were measured using a human MMP-3 Plex Ultrasensitive kit (Mesoscale, Rockville, MD); transforming growth factor (TGF)-β1, TGF-β2, and TGF-β3 using a human U-PLEX TGF-β Combo kit (Mesoscale, Rockville, MD); and basic fibroblast growth factor (bFGF) using a Human V-PLEX bFGF kit (Mesoscale, Rockville, MD). SF levels of C-terminal telopeptides of type 2 collagen (CTX-2) and Aggrecan (ACG) were analyzed by enzyme-linked immunosorbent assay (ELISA) (MyBiosource, VersaMax™). All samples were run in duplex and performed according to the manufacturer’s instructions. Protein values below lower limit of detection (LLOD) were replaced by a value of ½ LLOD for statistical analysis. The percentage of proteins below LLOD and the coefficient of variation (CV) are presented in Additional file 2: Supplementary 1.

### Clinical follow-up 3 and 12 months after ankle surgery

After 3 and 12 months, the patients were invited to clinical and radiographic examination with plain X-ray and low-dose weight-bearing cone beam CT (WBCT) [14]. The following parameters were recorded: ankle swelling, VAS pain score (at rest and on activity), time of return to work after surgery, degree of mobilization (4-point scale), EQ-5D-5L index score Kellgren-Lawrence score for severity of osteoarthritis (grades 0–4, where 0 = healthy ankle) [15], the American Orthopedic Foot and Ankle Society (AOFAS) score (ranging 0-100, where 100 = healthy ankle) [16], and the Danish version of Foot Function Index (FFI-DK) (ranging 0–230, with a high score indicating poor outcome) [17]. All examinations were performed by the same surgeon (TMP) at both follow-ups.

### Evaluation of post-operative fracture malreduction, Kellgren-Lawrence score, and joint space narrowing

**Fig. 2:**
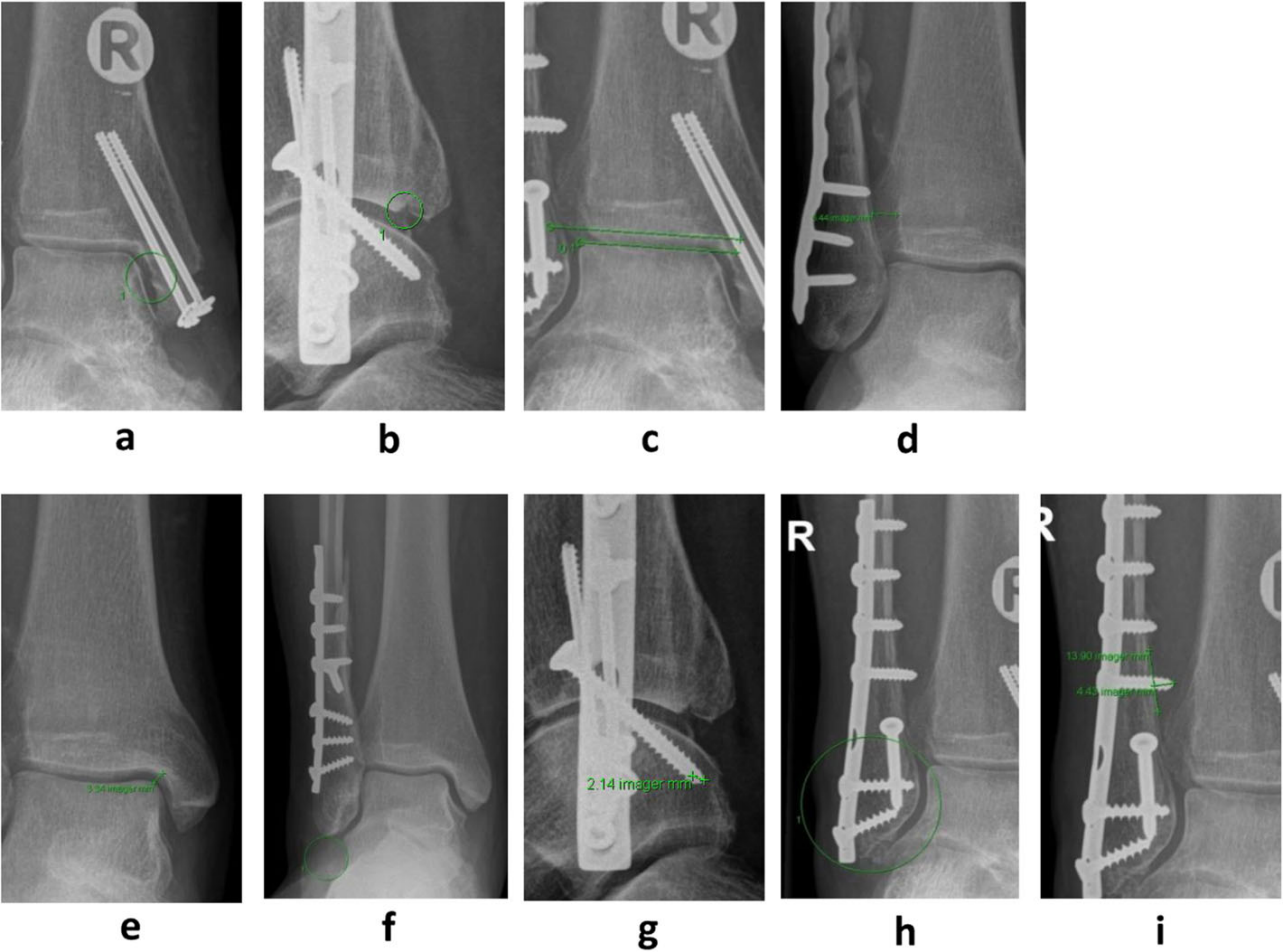
Fracture reduction criteria measured on post-operative X-ray 3 months after ankle surgery. **2a** Medial step-off in mortise view. **2b** Posterior step-off: only included if more than 20% of the articulating surface was affected in lateral view. **2c** Tibiotalar tilt: measured in mortise view as the angle between the tibial plafond and the talar joint line. **2d** Tibiofibular overlap: defined as the distance between the medial edge of the fibula and incisura fibularis and measured 10 mm proximal to the tibial plafond in mortise view. **2e** Oblique medial clear space: measured between the inferior-medial corner of the tibial plafond and the superior-medial corner of the talus in mortise view. **2f** Dime sign: fibula is shortened and reported as “positive” dime sign if the “ball” is broken in mortise view. **2g** Lag screw surpassing the bone cortex (bicortical): the distance was measured perpendicular to the cortical line. **2h** Distal fibular screws: defined as “positive” if any of the 3 distal screws was bicortical in any projection. **2i** Proximal fibular screws: the distance was measured perpendicular to the cortical line of the most penetrating screw in any projection. All measurements were reported in mm or positive/negative

**Fig. 3:**
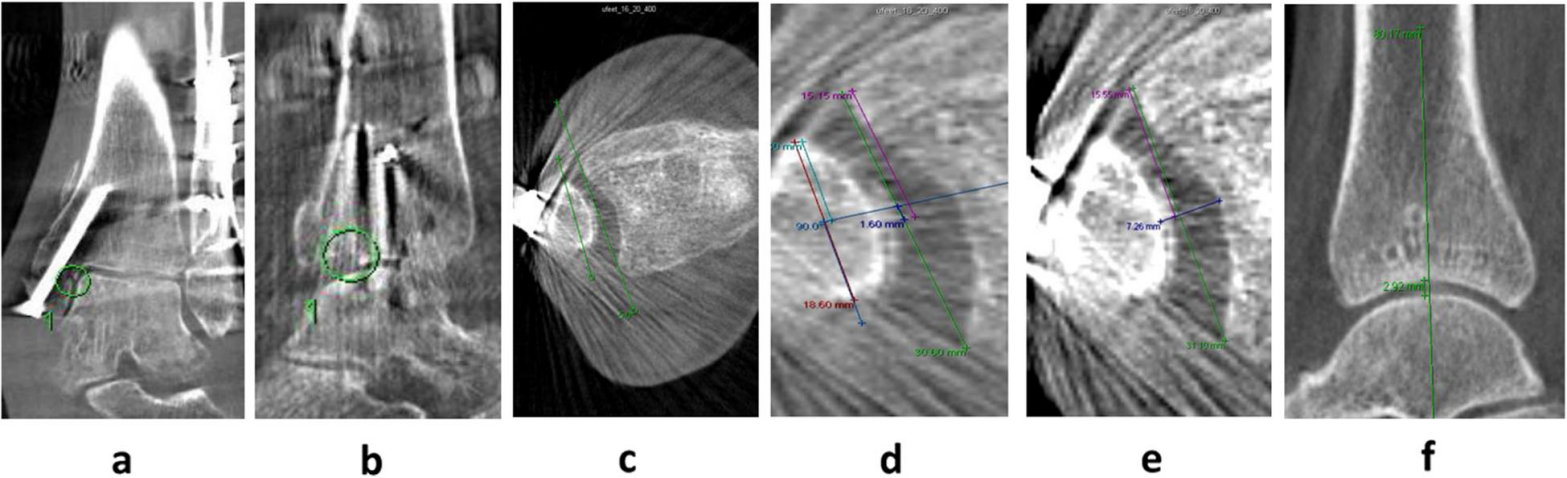
Fracture reduction criteria measured on post-operative WBCT 3 months after ankle surgery. **a** Medial step-off: measured in the coronal plane on the slide with the highest degree of step-off. **3b** Posterior step-off: measured in the sagittal plane on the slide with the highest grade of step-off (only included if more than 20% of the articulating surface was affected). **3c** Fibular rotation: the deviation of rotation of the fibula relative to the tibia was measured as the angle of a line between the posterior and anterior tibial tubercles, and a line between the anterior and posterior tubercle of fibula. Measurements of **3c**, **3d**, and **3e** were always performed 10 mm (10 slides) proximal from the beginning of the distal tibial articular surface in coronal plane. **3d** Fibular anteroposterior translation: fibular translational deviation anteriorly or posteriorly relative to tibia. The anterior or posterior deviation is being measured with a line (light blue) perpendicular to the midpoint (turquoise line) of the anterior and posterior fibular tubercle (red line). The point where the light blue line crosses the line between the anterior and posterior tibial tubercle (green line) relative to the respective midpoint of this (green) line defines the anterior and posterior translational deviation (dark blue line). **3e** Tibiofibular widening: the distance between the medial aspect of the cortex of the fibula and the lateral aspect of the cortex of the tibia. This distance was measured at an angle perpendicular to the midpoint between the anterior and posterior tibial tubercle. **3f** Joint space narrowing: defined as the difference in joint space at 3 months minus 12 months. Measured in sagittal plane as the distance between the articular surface of talus and tibia at the central line of tibia

Fracture malreduction was based on 9 criteria on plain X-ray (medial step-off, posterior step-off, tibiotalar tilt, tibiofibular (TF) overlap, oblique medial clear space, dime sign, lag screw, distal fibula screws, and proximal fibular screws) (Fig. 2) and 5 criteria on WBCT (medial step-off, posterior step-off, fibular rotation, fibular anteroposterior translation, and tibiofibular widening) (Fig. 3). All criteria have previously been defined as important for fracture reduction during ankle surgery [18, 19]. Furthermore, peri-operative X-rays from all patients were approved by orthopedic specialists after surgery. The measurements were performed in GE Web PACS 3.0 based on 3 months follow-up and were performed twice by two independent investigators (EBK/TMP) with 2 weeks between each measurement. Inter-observer and intra-observer reliability were determined using the intra-class correlation coefficient (ICC).

The Kellgren-Lawrence score was measured on X-ray 12 months after surgery and joint space narrowing was defined as the difference in joint space measured at 3 months and 12 months on WBCT (Fig. 3).

### Statistical methods

Quantile-quantile (q-q)-plot tests of nearly all cytokines, radiographic criteria, and clinical outcomes indicated a parametric pattern. The correlation of cytokine levels and radiographic criteria versus clinical outcomes was performed using mixed effects logistic regression models for longitudinal data adjusted for age, sex, BMI, and AO fracture classification. Correlation of fracture classification versus clinical outcome and protein levels was performed using Spearman’s rho correlation coefficient. Results are presented as means with standard deviations, and a *p* value < 0.05 was considered significant. All statistical analyses were performed using STATA MP 16.

## Results

Of the 46 patients with ankle fracture who were recruited during the study period, four patients were excluded during the follow-up (Fig. 1). The 42 patients included in the final analysis comprised 18 males and 24 females with a mean age of 42.7 ± 3.6 years. Most of the ankle fractures were type 44B (*n* = 26) and 44C (*n* = 14). The most common surgical treatments were open reduction internal fixation (ORIF) with plate and screws with or without tight rope or syndesmotic screw (*n* = 37). SF was collected on average 4.5 days after fracture, ranging from 0 to 13 days (Table 1). Nearly all proteins were elevated in fractured ankles compared to healthy contralateral ankles (Additional file 2: Supplementary 2).

**Table 1:** Baseline characteristics of 42 patients with ankle fracture

Sex—n (%)	
Male	18 (42.9%)
Female	24 (57.1%)
Age—mean (SD)	42.7 (3.6)
BMI—mean (SD)	27.5 (3.6)
ASA score—*n* (%)	
1	20 (47.6%)
2	22 (52.4%)
3	0 (0%)
4	0 (0%)
Fracture side—*n* (%)	
Left	16 (38.1%)
Right	26 (61.9%)
AO classification—*n* (%)	
44A	2 (4.8%)
44B	26 (61.9%)
44C	14 (33.3%
Time of SF collection—mean (min.–max.)	4.5 days (0–13 days)
Method of operation—n (%)	
Plate and/or screws—only lateral malleolus	3 (7.1%)
Plate and/or screws—only medial malleolus	4 (9.5%)
Plate and/or screws—medial and lateral malleolus	16 (38.1%)
Plate and screws—medial and/or lateral malleolus + tight rope	6 (14.3%)
Plate and screws—medial and/or lateral malleolus + syndesmotic screw	8 (19.0%)
Other	5(11.9%)

*Abbreviation*: *AO* Arbeitsgemeinschaft für Osteosynthesefragen, *SD* Standard deviation, *BMI* body mass index, *ASA* American Society of Anesthesiologists Classification

Overall, the clinical outcome improved considerably from 3 to 12 months after surgery (Table 2). At 12 months, however, patients still had pain in the ankle on activity (VAS = 2.5 ± 2.4), 56.1% had not achieved normal mobilization, and 16.7% had not returned to work. Analysis of functional outcomes revealed a mean AOFAS score of 80.8 ± 13.1, a FFI-DK score of 45.5 ± 46.9 and EQ-5D-5L score of 0.75 ± 0.16 (Table 2).

**Table 2:** Clinical outcomes 3 and 12 months after surgery for 42 patients with ankle fracture

AOFAS score—3 months (mean ± SD)	72.8 ± 13.6
AOFAS score—12 months (mean ± SD)	80.8 ± 13.1
FFI-DK score—3 months (mean ± SD)	71.7 ± 48.1
FFI-DK score—12 months (mean ± SD)	45.5 ± 46.9
EQ-5D-5L index score 3 months (mean ± SD)	0.69 ± 0.13
EQ-5D-5L index score 12 months (mean ± SD)	0.75 ± 0.16
Kellgren-Lawrence score—12 months	1.5 ± 0.9
Joint space narrowing after–12 months (mm)	− 0.13 ± 0.48
VAS pain score—3 months at rest (mean ± SD)	1.9 ± 1.8
VAS pain score—3 months on activity (mean ± SD)	3.6 ± 2.6
VAS-score—12 months at rest (mean ± SD)	1.0 ± 1.4
VAS-score—12 months on activity (mean ± SD)	2.5 ± 2.4
Ankle swelling—3 months from surgery (mean ± SD) (cm)	2.2 ± 1.3
Ankle swelling—12 months from surgery (mean ± SD) (cm)	1.2 ± 1.0
Returned to work—3 months from surgery (yes/no)	(67.7%/32.5%)
Returned to work—12 months from surgery (yes/no)	(83.3%/16.7%)
Mobilization after 3 months	
1: Still need assistive devices	15.0%
2: Can walk without assistive devices	7.5%
3: Can walk more than 500 m without assistive devices	70.0%
4: Returned to normal mobilization	7.5%
Mobilization after 12 months	
1: Still need assistive devices	0.0%
2: Can walk without assistive devices	0.0%
3: Can walk more than 500 m without assistive devices	56.1%
4: Returned to normal mobilization	43.9%

AOFAS (range 0–100 points, where 100 = healthy ankle), FFI-DK (range 0–230 points, a high score indicating poor outcome), EQ-5D-5L index score (range − 0.224 to 1, where 1 corresponds to full health and negative values reflect health states worse than death). Kellgren-Lawrence score (grade 0–4, a higher score reflecting more severe osteoarthritis)

AO fracture classification showed no significant correlation with any of the clinical outcomes at 12 months follow-up (Additional file 2: Supplementary 3). Furthermore, AO fracture classification showed significant correlation with only 5 of the 22 protein levels analyzed (IL-4, IL-6, IL-12p70, MMP-1, and MMP-3) (Additional file 2: Supplementary 4).

### Correlation between fracture reduction quality and clinical outcomes after ankle surgery

We found statistically significant correlations between several radiographic criteria and clinical outcomes at 12 months after surgery (Table 3).

**Table 3:** Correlation between fracture reduction quality (from plain x-ray and weight-bearing CT (WBCT)) and clinical outcomes 12 months after surgery for ankle fracture (*n* = 42)

	Kellgren-Lawrence score *p* value (coef.)	Joint space narrowing on WBCT *p* value (coef.)	FFI-DK score *p* value (coef.)	AOFAS score *p* value (coef.)	EQ-5D-5L **i**ndex score *p* value (coef.)
X-ray criteria					
*Medial step-off*	0.02 (− 0.09)	–	–	–	–
*Posterior step-off*	–	–	–	0.04 (10.4)	–
*Tibiotalar tilt*	–	0.03 (0.22)	–	–	–
*Tibiofibular overlap*	–		0.02 (11.2)	0.0004 (− 3.6)	–
*Oblique medial clear space*	–	0.007 (0.18)	–	–	–
*Dime sign*	–		0.008 (48.6)	–	–
*Lag screw*	–	0.003 (0.28)		–	–
*Distal fibula screws*	–	–	–	–	–
*Proximal fibular screws*	–	–	–	–	–
WBCT criteria	–			–	–
*Medial step-off*	–	–	0.03 (− 42.7)	–	–
*Posterior step-off*	–	–	0.046 (− 17.3)	–	–
*Fibular rotation*	–	–	-	0.04 (0.7)	–
*Tibiofibular widening*	–	–	0.04 (10.7)	0.02 (3.3)	0.02 (− 0.04)
*Fibular anteroposterior translation*	–	–	–	–	–

*Coef*: coefficient value (ranges from − 1 to 1 and indicates that a change of 1 unit in the variable will result in a change of x units in the outcome score). Only *p* values below 0.05 are reported

“–” indicates no statistical significance. Adjusted for age, sex, body mass index, and AO fracture classification

For plain X-ray criteria, a positive correlation was found between posterior step-off and AOFAS score (*p* = 0.04), between TF overlap and FFI-DK score (*p* = 0.02), and between dime sign and FFI-DK score (*p* = 0.008). However, a negative correlation was observed between TF overlap and AOFAS score (*p* = 0.0004) and between medial step-off and Kellgren-Lawrence score (*p* = 0.02). Tibiotalar tilt was positively correlated to joint space narrowing at 3 months (*p* = 0.01) and 12 months (*p* = 0.03).

For WBCT criteria, TF widening was correlated to FFI-DK score (*p* = 0.04), AOFAS score (*p* = 0.02), and EQ-5D-5L index score (*p* = 0.02). Only FFI-DK was significantly negatively correlated to medial step-off (*p* = 0.03) and posterior step-off (*p* = 0.046) on WBCT (Additional file 2: Supplementary 6 and 7).

Intra-observer reliability (ICC) was good for all X-ray criteria combined (0.86) and for all WBCT criteria combined (0.75). Inter-observer reliability was moderate for all X-ray criteria combined (0.68) and poor for all WBCT criteria combined (0.31). ICC values for the individual radiographic criteria are provided in Additional file 2: Supplementary 5.

### Correlation between protein levels in ankle synovial fluid and clinical outcomes after ankle surgery

Table 4 presents an overview of protein levels and clinical outcomes 12 months after ankle surgery. A negative correlation was observed between Kellgren-Lawrence score and IL-6 (*p* = 0.04), IFN-y (0.048), and IL-4 (*p* = 0.03). TGF-β2 was negatively correlated with FFI-DK score (*p* = 0.009), while IL-1 β was positively correlated with AOFAS score (*p* = 0.03) and EQ5D-5L index score (*p* = 0.02). The remaining proteins showed no statistical correlations.

**Table 4:** correlation between protein levels in synovial fluid and clinical and radiographic outcomes 12 months after surgery for ankle fracture (*n* = 42)

	Protein levels	Kellgren-Lawrence score *p* value (coef.)	Narrowing in joint space *p* value (coef.)	FFI-DK *p* value (coef.)	AOFAS *p* value (coef.)	EQ5D-5L *p* value (coef.)
Pro-inflammatory	IL-1α	–	–	–	–	–
	IL-1β	–	–	–	0.03 (0.17)	0.02 (0.002)
	IL-2	–	–	–	–	–
	IL-6	0.04 (− 0.0004)	–	–	–	–
	IL-8	–	–	–	–	–
	IL-12p70	–	–	–	–	–
	TNF-α	–	–	–	–	–
	TNF-β		–	–	–	–
	IFN-y	0.048 (− 0.01)	–	–	–	–
	MMP-1	–	–	–	–	–
	MMP-3	–	–	–	–	–
	MMP-9	–	–	–	–	–
Anti-inflammatory	IL-1RA	–	–	–	–	–
	IL-4	0.03 (− 0.38)	–	–	–	–
	IL-10	–	–	–	–	–
	IL-13	–	–	–	–	–
Cartilage degradation	ACG	–	–	–	–	–
	CTX2	–	–	–	–	–
Metabolic	b-FGF	–	–	–	–	–
	TGF-β1	–	–	–	–	–
	TGF-β2	–	–	0.009 (− 0.5)	–	–
	TGF-β3	–	–	–	–	–

*Coef*: coefficient value (ranges from − 1 to 1 and indicates that a change of 1 unit in the variable will result in a change of x units in the outcome score). Only *p* values below 0.05 are reported

“–” indicates no statistical significance. Adjusted for age, sex, body mass index, and AO fracture classification.

## Discussion

In this study, we investigated nine criteria on plain X-ray and five criteria on weight-bearing CT, thus covering many of the radiographic al parameters hypothesized to be important for clinical outcome after ankle surgery [6, 18, 19]. We found that TF overlap measured on X-ray and TF widening measured on WBCT were especially important for clinical outcome at 12 months after ankle surgery. Malreduction of TF overlap and TF widening reflect insufficiency of the syndesmosis and has previously been correlated with worse functional outcome [7, 20]. Fibular rotation, oblique medial clear space, and fibular anteroposterior translation have also been described as reflecting insufficiency of syndesmosis reduction [21], but these criteria did not significantly correlate with clinical outcomes in our study. This may be due to the longer follow-up (2 years) and the use of Olerud/Molander questionnaires in the previous study. We found that TF tilt measured X-ray, correlated with ankle joint space narrowing from 3 to 12 months, possibly indicating that cartilage degradation is influenced by the injury and the inflammatory response. In contrast, TF tilt was not significantly correlated with any functional outcome, possibly due to the relatively short follow-up of 12 months.

While the posterior step-off criterion has also been reported to be important for functional outcome after ankle surgery [7, 20], we found no significant correlation between posterior step-off and clinical outcomes at 12 months post-surgery. This might be explained by the difference in follow-up time. Additionally, malreduction of more than two radiographic criteria in comminuted ankle fractures may worsen the functional outcomes as described by Pettrone et al. (1983). These authors found that the presence of a higher number of malreduction criteria on post-operative X-rays was correlated with poorer functional outcomes [6].

The clinical importance of fracture malreduction evaluated by CT has been the subject of extensive research, and several studies report it to be superior to evaluation by plain X-ray [21]. To the best of our knowledge, no previous study has correlated fracture malreduction on CT and functional outcomes after ankle surgery. However, Ntalos et al. (2018) used magnetic resonance imaging to evaluate fracture malreduction and found no significant correlation between malreduction and either AOFAS score or SF-36 score after a follow-up of 34.5 months. In our study, no malreduction criteria on plain X-ray were correlated with AOFAS, but TF widening on WBCT was associated with poorer quality of life (EQ-5D-5L) and FFI-DK scores. This finding may indicate the clinical impact of using three-dimensional scans with weight-bearing in the management of ankle fractures.

The initial inflammatory cascade in the joint space after acute ankle fracture has recently been extensively investigated due to the hypothesis that the presence of pro-inflammatory cytokines in the joint leads to development of PTOA. Several cytokines (such as IL-1β, lL-6, IL-8, IL-10, and TNF-α) and MMPs have been reported to be elevated in the joint space of patients with osteoarthritis. We found an elevation of all these proteins in acute intra-articular ankle fractures compared to healthy contralateral ankle joints, and for some proteins (such as IL-6, IL-8, IL-1RA, and MMP1), the elevation was more than 100-fold. Adams et al. (2017) reported that concentrations of IL-6, IL-8, MMP1, MMP2, and MMP3 were still elevated 6 months after ankle fracture [22]. We evaluated the levels of 22 proteins in fractured ankles and their correlation with functional outcomes 12 months after ankle surgery. We found that the Kellgren-Lawrence score correlated with IL-6, INF-γ, and IL-4 levels and that IL-2 levels correlated with AOFAS score and EQ-5D-5L index score. There was not a rational pattern in these correlations, however. For instance, elevated IL-1β levels post-injury correlated positively with AOFAS score at 12 months after surgery, but IL-1β are a pro-inflammatory cytokine and should theoretically affect AOFAS negatively. This suggests that the synovial inflammatory response following ankle fracture might just be a phenomenon of natural repair with limited short-term significance for functional outcomes. A greater effect of these cytokines on the joint cartilage may not be clinically present after 12 months however, and a longer follow-up may be necessary to clarify this potential association.

Our study has several limitations. First of all, we had a relatively small sample size, and the follow-up was only 12 months after surgery. However, we believe that the clinical impact of fracture malreduction is likely to be present after 12 months. Furthermore, a statistically significant correlation between cytokine levels and clinical outcomes might not be transferable to clinically relevant significance. In addition, the dynamic process of inflammation may affect cytokine levels after injury as illustrated in Additional file 2: Supplementary 8. However, we found that most of the cytokine levels in the fractured ankles did not correlate with the length of time between injury and SF aspiration (IL-1β, IL-2, IL-8, TNF-α, TNF-β, IL-1Ra, IL-10, IL-13, ACG, CTX-2 TGF-β1, and TGF-β2. This explorative study may be the first to report associations between functional outcomes after ankle surgery and the quality of fracture reduction and levels of inflammatory cytokines and metabolic proteins.

## Conclusions

In conclusion, we found that malreduction of tibiofibular overlap, tibiofibular tilt, and tibiofibular widening was associated with worse scores on functional outcomes. Elevation of inflammatory cytokines after acute ankle fracture was generally not correlated with functional outcomes at 12 months. Further studies with longer follow-up may be necessary to identify potential associations. This study provides additional information to the orthopedic surgeon when deciding which fracture reduction criteria to prioritize during surgery. The study also emphasizes that more research is needed before anti-inflammatory medication is used as a supplement to surgery in the treatment of ankle fractures.

## Supplementary Information

**Additional file 1.** STROBE Statement—Checklist of items that should be included in reports of cohort studies.

**Additional file 2: Supplementary 1.** LLOD and CV values of synovial fluid in fractured and contralateral ankle joints. **Supplementary 2**. Cytokine levels in fractured ankles compared to healthy contralateral ankles. **Supplementary 3.** Correlation between AO fracture classification and clinical outcomes 12 months after ankle surgery (n=42). **Supplementary 4.** Correlation between AO fracture classification and protein levels in fractured ankles 12 months after ankle surgery (n=42). **Supplementary 5.** Inter- and intra-observer reliability for fracture reduction criteria. **Supplementary 6.** Correlation between fracture reduction quality (from plain x-ray and weight-bearing CT (WBCT) and clinical outcomes 3 months after surgery for ankle fracture (n=42). **Supplementary 7.** Correlation between protein levels in synovial fluid and clinical and radiographic outcomes 3 months after surgery for ankle fracture (n=42). **Supplementary 8.** Correlation between protein levels and time after injury (0-13 days).

## Data Availability

All data are hosted online at OPEN (Open Patient data Explorative Network) and are available from the corresponding author on reasonable request and approval from the Regional Committees on Health Research Ethics for Southern Denmark and the National Danish Data Protection Agency https://www.sdu.dk/da/om_sdu/institutter_centre/klinisk_institut/forskning/forskningsenheder/open.Aspx.

## Abbreviations

ACG: Aggrecan
AO: Arbeitsgemeinschaft für Osteosynthesefragen
AOFAS: American Orthopedic Foot and Ankle Society
ASA: American Society of Anesthesiologists Classification
bFGF: Basic fibroblast growth factor
BMI: Body mass index
CT: Computed tomography
CTX-2: C-terminal telopeptides of type 2 collagen
CV: Coefficient of variation
ELISA: Enzyme-linked immunosorbent assay
EQ-5D: EuroQol-5 dimension
FFI-DK: The Danish version of Foot Function Index
ICC: Intra-class correlation coefficient
IFN-y: Interferon gamma
IL: Interleukin
LLOD: Below lower limit of detection
MMP: Matrix metalloproteinase
PTOA: Post-traumatic osteoarthritis
Qq: Quantile-quantile
RPM: Revolutions per minute
SF: Synovial fluid
TF: Tibiofibular
TGF: Transforming growth factor
TNF: Tumor necrosis factor
VAS: Visual analog scale
WBCT: Weight-bearing computed tomography
