# Supplementary material for: Association of acute inflammatory cytokines, fracture malreduction, and functional outcome 12 months after intra-articular ankle fracture—a prospective cohort study of 46 patients with ankle fractures
Source: J Orthop Surg Res. 2021 May 25;16:338. doi: 10.1186/s13018-021-02473-8 (PMC8146632; doi:10.1186/s13018-021-02473-8)
Supplement: Supplementary file 2 — Additional file 2: Supplementary 1. LLOD and CV values of synovial fluid in fractured and contralateral ankle joints. Supplementary 2. Cytokine levels in fractured ankles compared to healthy contralateral ankles. Supplementary 3. Correlation between AO fracture classification and clinical outcomes 12 months after ankle surgery (n=42). Supplementary 4. Correlation between AO fracture classification and protein levels in fractured ankles 12 months after ankle surgery (n=42). Supplementary 5. Inter- and intra-observer reliability for fracture reduction criteria. Supplementary 6. Correlation between fracture reduction quality (from plain x-ray and weight-bearing CT (WBCT) and clinical outcomes 3 months after surgery for ankle fracture (n=42). Supplementary 7. Correlation between protein levels in synovial fluid and clinical and radiographic outcomes 3 months after surgery for ankle fracture (n=42). Supplementary 8. Correlation between protein levels and time after injury (0-13 days). [file 13018_2021_2473_MOESM2_ESM.docx]

| supplementary 1: LLOD and CV values of synovial fluid in fractured and contralateral ankle joints | | | | | |
| --- | --- | --- | --- | --- | --- |
|  |  | **Fractured ankles (SF)**  **CV and LLOD** | | **Contralateral ankles (SF)**  **CV and LLOD** | |
|  |  | Percent of CV  above 20% | Percent below  LLOD | Percent of CV  above 20% | Percent below  LLOD |
| Pro-inflammatory | IL-1α | 8.5 | 83.0 | 80.5 | 90.2 |
|  | IL-1β | 6.4 | 8.5 | 14.6 | 90.2 |
|  | IL-2 | 12.7 | 23.4 | 8.5 | 97.6 |
|  | IL-6 | 4.3 | 0 | 2.4 | 90.2 |
|  | IL-8 | 4.3 | 0 | 0 | 12.2 |
|  | IL-12p70 | 14.9 | 10.6 | 4.9 | 95.1 |
|  | TNF-α | 17.0 | 6.4 | 7.3 | 97.6 |
|  | TNF-β | 25.5 | 97.9 | 34.1 | 100 |
|  | IFN-y | 14.9 | 23.4 | 26.8 | 100 |
|  | MMP-1 | 2.4 | 0 | 2.8 | 50 |
|  | MMP-3 | 0 | 0 | 8.3 | 0 |
|  | MMP-9 | 0 | 0 | 16.7 | 55.6 |
| Anti-inflammatory | IL-1RA | 10.6 | 0 | 17.1 | 34.1 |
|  | IL-4 | 17.0 | 14.9 | 19.5 | 4.9 |
|  | IL-10 | 10.6 | 4.3 | 7.3 | 92.7 |
|  | IL-13 | 31.9 | 4.3 | 9.8 | 100 |
| Cartilage  Degradation | ACG | 4.8 | 0 | 9.1 | 6.1 |
|  | CTX-2 | 21.1 | 18.4 | 14.8 | 18.5 |
| Metabolic | bFGF | 0 | 0 | 2.9 | 0 |
|  | TGF-β1 | 4.3 | 0 | 2.4 | 7.3 |
|  | TGF-β2 | 0 | 4.3 | 7.3 | 63.4 |
|  | TGF-β3 | 0 | 53.2 | 22.0 | 100 |
| LLOD: Lower limit of detection, CV: Coefficients of variation, SF: synovial fluid | | | | | |

| Supplementary 2: Cytokine levels in fractured ankles compared to healthy contralateral ankles | | | | | |
| --- | --- | --- | --- | --- | --- |
|  | **Protein levels** | **Fractured ankles (pg/mL)**  **Mean ± SD** |  | **Contralateral ankles (pg/mL)**  **Mean ± SD** | **Cytokine ratio**  **(fractured/contralateral)** |
| Pro-inflammatory | IL-1α | 0.0067 ± 0.0071 |  | 0.006 ± 0.004 | 1.1 |
|  | IL-1β | 10.8 ±23.0 |  | 0.25 ±0.59 | 47.4 |
|  | IL-2 | 4.2 ±3.6 |  | 0.51 ±0.11 | 8.8 |
|  | IL-6 | 1404.0 ±746.3 |  | 4.0 ±23.1 | 370 |
|  | IL-8 | 729.2 ±608.6 |  | 729.2 ±608.6 | 233 |
|  | IL-12p70 | 11.7 ±14.5 |  | 0.92 ±0.44 | 12.7 |
|  | TNF-α | 11.6 ±13.9 |  | 0.52 ±0.34 | 23.8 |
|  | TNF-β | 0.00004 ±0.00002 |  | 0.0005 ±0.00001 | 0.9 |
|  | IFN-y | 33.1 ±30.1 |  | 3.8 ±0.71 | 9.4 |
|  | MMP-1 | 178970 ±308807 |  | 1592.5 ± 3778.1 | 126 |
|  | MMP-3 | 412044 ±423151 |  | 20930 ± 53188 | 21.7 |
|  | MMP-9 | 161240 ±153557 |  | 4046 ±8655 | 32.6 |
| Anti-inflammatory | IL-1RA | 2130.1 ± 1786.9 |  | 6.8 ± 13.4 | 314 |
|  | IL-4 | 0.86 ±0.81 |  | 0.07 ±0.04 | 13.1 |
|  | IL-10 | 124.7 ±583.5 |  | 5.3 ±24.2 | 26.6 |
|  | IL-13 | 55.7 ±37.9 |  | 4.4 ±0.3 | 13.7 |
| Cartilage degradation | ACG | 2099.9 ±1212.6 |  | 1268.9 ±632.7 | 1.6 |
|  | CTX-2 | 297.7 ±235.1 |  | 152.0 ±56.6 | 1.8 |
| Metabolic | bFGF | 167.5 ±313.5 |  | 59.4 ±73.9 | 1.5 |
|  | TGF-β1 | 3735.4 ±3746.8 |  | 498.8 ±715.5 | 7.3 |
|  | TGF-β2 | 50.8 ±28.2 |  | 29.2 ±34.6 | 1.8 |
|  | TGF-β3 | 5.6 ± 5.2 |  | 5.5 ±1.8 | 1.0 |
| SD: Standard deviation.  Nearly all proteins were more elevated in fractured ankle joints compared to healthy contralateral ankle joints. | | | | | |

| supplementary 3: Correlation between AO fracture classification and clinical outcomes 12 months after ankle surgery (n=42) | |
| --- | --- |
| Clinical outcomes at 12 months | **P-value (rho)** |
| Ankle swelling | 0.73 (-0.04) |
| VAS pain score - at rest | 0.30 (-0.12) |
| VAS pain score - on activity | 0.59 (-0.06) |
| FFI-DK score | 0.37 (-0.10) |
| AOFAS score | 0.29 (0.12) |
| EQ-5D-5L index score | 0.37 (-0.10) |
| Kellgren Lawrence score | 0.35 (0.1509) |
| Narrowing in joint space | 0.49 (-0.1149) |
| AO: Arbeitsgemeinschaft für Osteosynthesefragen. | |

| supplementary 4: Correlation between AO fracture classification and protein levels in fractured ankles 12 months after ankle surgery (n=42) | | | |
| --- | --- | --- | --- |
|  | | **Protein levels** | **P-value (rho)** |
| Pro-inflammatory | IL-1α | | 0.18 (-0.16) |
|  | IL-1β | | 0.15 (0.17) |
|  | IL-2 | | 0.19 (0.16) |
|  | IL-6 | | **0.02 (0.27)** |
|  | IL-8 | | 0.34 (0.11) |
|  | IL-12p70 | | **0.02 (0.27)** |
|  | TNF-α | | 0.24 (0.15) |
|  | TNF-β | | 0.91 (0.01) |
|  | IFN-y | | 0.14 (0.19) |
|  | MMP-1 | | **<0.000 (0.55)** |
|  | MMP-3 | | **0.02 (0.29)** |
|  | MMP-9 | | 0.27 (-0.14) |
| Anti-inflammatory | IL-1RA | | 0.09 (0.27) |
|  | IL-4 | | **0.04 (0.33)** |
|  | IL-10 | | 0.07 (0.29) |
|  | IL-13 | | 0.11 (0.25) |
| Cartilage degradation | ACG | | 0.62 (0.08) |
|  | CTX-2 | | 0.16 (0.23) |
| Metabolic | bFGF | | 0.41 (-0.11) |
|  | TGF-β1 | | 0.15 (-0.19) |
|  | TGF-β2 | | 0.57 (0.08) |
|  | TGF-β3 | | 0.37 (0.12) |
| Data in bold indicate a significant correlation (p < 0.05).  Protein levels: Protein level in the fractured joints minus the levels in the healthy contralateral joints. Rho indicates if the correlation is positive or negative. | | | |

| Supplementary 5: Inter- and intra-observer reliability for fracture reduction criteria | | |
| --- | --- | --- |
|  | **Inter-observer**  **ICC value** | **Intra-observer**  **ICC value** |
| X-ray criteria |  |  |
| Medial step-off | 0.00 | 0.44 |
| Posterior step-off | 1.00 | 0.89 |
| Tibiotalar tilt | 0.40 | 0.80 |
| Tibiofibular overlap | 0.82 | 0.88 |
| Oblique medial clear space | 0.89 | 0.93 |
| Dime sign | 0.66 | 1.00 |
| Lag screw | 0.93 | 0.93 |
| Distal fibular screws | 0.55 | 1.00 |
| Proximal fibular screws | 0.88 | 0.90 |
| Average of all X-ray criteria | 0.68 | 0.86 |
| WBCT criteria |  |  |
| Medial step-off | 0.31 | 0.39 |
| Posterior step-off | 0.75 | 0.94 |
| Fibular rotation | 0.10 | 0.78 |
| Fibular antero-posterior translation | -0.19 * | 0.85 |
| Tibiofibular widening | 0.56 | 0.81 |
| Average of all WBCT criteria | 0.31 | 0.75 |
| Abbreviation: ICC: intraclass correlation coefficient (values <0.5 indicate poor reliability, values from 0.5 to 0.75 indicate moderate reliability, values from 0.75 to 0.9 indicate good reliability, and values >0.90 indicate excellent reliability).  * ICC value was reported as negative in STATA due to the definition of fibular anterior translation (anterior to the defined midpoint was reported as positive, and posterior was reported as negative). | | |

| supplementary 6 : correlation between fracture reduction quality (from plain x-ray and weight-bearing CT (WBCT) and clinical outcomes 3 months after surgery for ankle fracture (n=42) | | | |
| --- | --- | --- | --- |
|  | **FFI-DK score**  P-value (coef.) | **AOFAS score**  P-value (coef.) | **EQ-5D-5L index score**  P-value (coef.) |
| X-ray criteria |  |  |  |
| Medial step-off | **-** | **-** | **-** |
| Posterior step-off | **-** | **-** | 0.001(-0.12) |
| Tibiotalar tilt | 0.01 (23.3) | 0.048 (-5.1) |  |
| Tibiofibular overlap | **-** | **-** | **-** |
| Oblique medial clear space | **-** | **-** | **-** |
| Dime sign | 0.003 (54.1) | 0.02 (-11.6) | **-** |
| Lag screw | **-** | **-** |  |
| Distal fibula screws | **-** | **-** | **-** |
| Proximal fibular screws | **-** | **-** | **-** |
| WBCT criteria |  |  |  |
| Medial step-off | **-** | **-** | **-** |
| Posterior step-off |  |  |  |
| Fibular rotation | **-** | 0.03 (0.74) | - |
| Tibiofibular widening | **-** | **-** | **-** |
| Fibular anteroposterior translation | - | - | - |
| Coef: Coefficient value (ranges from -1 to 1 and indicates that a change of 1 unit in the variable will result in a change of x units in the outcome score). Only p-values below 0.05 are reported. “-“ indicates no statistical significance. Adjusted for age, sex, body mass index, and AO fracture classification. | | | |

| Supplementary 7 : correlation between protein levels in synovial fluid and clinical and radiographic outcomes 3 months after surgery for ankle fracture (n=42) | | | | |
| --- | --- | --- | --- | --- |
|  | **Protein levels** | **FFI-DK score**  P-value (coef.) | **AOFAS score**  P-value (coef.) | **EQ5D-5L index score**  P-value (coef.) |
| Pro-inflammatory | IL-1α | - | - | - |
|  | IL-1β | - | - | - |
|  | IL-2 | - | - | - |
|  | IL-6 | - | - | - |
|  | IL-8 | - | - | - |
|  | IL-12p70 | - | - | - |
|  | TNF-α | - | - | - |
|  | TNF-β | - | - | - |
|  | IFN-y | - | - | - |
|  | MMP-1 | - | - | - |
|  | MMP-3 | - | - | - |
|  | MMP-9 | - | - | - |
| Anti-inflammatory | IL-1RA | - | - | - |
|  | IL-4 | - | - | - |
|  | IL-10 | - | - | - |
|  | IL-13 | - | - | - |
| Cartilage degradation | ACG | - | - | - |
|  | CTX2 | - | - | - |
| Metabolic | b-FGF | - | - | - |
|  | TGF-β1 | - | - | - |
|  | TGF-β2 | - | - | 0.005 (0.002) |
|  | TGF-β3 | - | - | - |
| Coef: Coefficient value (ranges from -1 to 1 and indicates that a change of 1unit in the variable will result in a change of x units in the outcome score). Only p-values below 0.05 are reported. “-“indicates no statistical significance. Adjusted for age, sex, body mass index, and AO fracture classification. | | | | |

| supplementary 8: Correlation between protein levels and time after injury (0-13 days) | | |
| --- | --- | --- |
|  | **Protein levels** | P**-value (rho)** |
| Pro-inflammatory | IL-1α | **0.03 (-0.318)** |
|  | IL-1β | 0.37 (-0.134) |
|  | IL-2 | 0.53 (-0.094) |
|  | IL-6 | **0.01 (-0.362)** |
|  | IL-8 | 0.91 (0.017) |
|  | IL-12p70 | **0.009 (-0.375)** |
|  | TNF-α | 0.52 (-0.095)) |
|  | TNF-β | 0.225 (-0.194) |
|  | IFN-y | **0.03 (-0.322)** |
|  | MMP-1 | **<0.0001 (0.683)** |
|  | MMP-3 | **0.0008 (0.505)** |
|  | MMP-9 | **0.003 (-0.448)** |
| Anti-inflammatory | IL-1RA | 0.25 (-0.170) |
|  | IL-4 | **0.03 (-0.319)** |
|  | IL-10 | 0.26 (-0.169) |
|  | IL-13 | 0.45 (-0.113) |
| Cartilage degradation | ACG | 0.47 (0.124) |
|  | CTX-2 | 0.52 (-0.110) |
| Metabolic | bFGF | **0.004 (-0.472)** |
|  | TGF-β1 | 0.06 (-0.317) |
|  | TGF-β2 | 0.46 (-0.126) |
|  | TGF-β3 | **0.0002 (0.578)** |
| Data in bold indicate a significant correlation (p < 0.05).  Rho indicates if the correlation is positive or negative. | | |
